# Genome-wide identification and characterization of laccase family members in eggplant (*Solanum melongena* L.)

**DOI:** 10.7717/peerj.12922

**Published:** 2022-02-21

**Authors:** Faxiang Wan, Linqing Zhang, Mengying Tan, Xiaohua Wang, Guang-Long Wang, Mengru Qi, Bingxin Liu, Jun Gao, Yu Pan, Yongqing Wang

**Affiliations:** 1 Faculty of Life Science and Food Engineering, Huaiyin Institute of Technology, Huaian, Jiangsu, The People’s Republic of China; 2 College of Horticulture and Landscape Architechture, Southwest University, Chongqing, The People’s Republic of China; 3 The Institute of Vegetable and Flower Research, Chongqing Academy of Agricultural Science, Chongqing, The People’s Republic of China

**Keywords:** Laccase, Expression patterns, Cold stress, Eggplant

## Abstract

Laccase, as a copper-containing polyphenol oxidase, primarily functions in the process of lignin, anthocyanin biosynthesis, and various abiotic/biotic stresses. In this study, forty-eight laccase members were identified in the eggplant genome. Only forty-two laccase genes from eggplant (*SmLACs*) were anchored unevenly in 12 chromosomes, the other six *SmLACs* were mapped on unanchored scaffolds. Phylogenetic analysis indicated that only twenty-five *SmLACs* were divided into six different groups on the basis of groups reported in *Arabidopsis*. Gene structure analysis revealed that the number of exons ranged from one to 13. Motif analysis revealed that SmLACs included six conserved motifs. In aspects of gene duplication analysis, twenty-one *SmLACs* were collinear with *LAC* genes from *Arabidopsis*, tomato or rice. Cis-regulatory elements analysis indicated many *SmLACs* may be involved in eggplant morphogenesis, flavonoid biosynthesis, diverse stresses and growth/development processes. Expression analysis further confirmed that a few *SmLAC*s may function in vegetative and reproductive organs at different developmental stages and also in response to one or multiple stresses. This study would help to further understand and enrich the physiological function of the *SmLAC* gene family in eggplant, and may provide high-quality genetic resources for eggplant genetics and breeding.

## Introduction

Laccase is a copper-containing polyphenol oxidase, belonging to the copper blue oxidase family. Moreover, laccases primarily catalyze the biological oxidation of a range of polyphenols with a concomitant reduction of molecular oxygen to water (Burke & Cairney, 2002; Mayer & Staples, 2002). Typically, laccase proteins contain three conserved domains of Cu-oxidase, Cu-oxidase_2 and Cu-oxidase_3 (Hoegger et al., 2010; N.Dwivedi et al., 2011).

The earliest laccases were extracted from the sap of the Japanese lacquer tree (Yoshida, 1883) and great progress has been made in the application of laccase research to areas such as in food processing industry, medical, health care, the design of biosensors and nanotechnology (Chauhan, Goradia & Saxena, 2017; Upadhyay, Shrivastava & Agrawal, 2016; Wang et al., 2015). Moreover, laccases have been found to be in microorganisms, insects and plants, and exhibited different functions on these organisms (Balasubramanian et al., 2016; Claus, 2003; Dittmer et al., 2004; Mccaig, Meagher & Dean, 2005; N.Dwivedi et al., 2011; Petr, 2006). In terms of microorganisms, they function mainly to decompose lignin. For example, all wood-decomposing fungi contain laccases (Leonowicz et al., 2001), but the detailed mechanism of delignification remains to be studied (Hoegger et al., 2010; Petr, 2006; Wang et al., 2017b). Apart from this function, fungal laccases are also involved in fruiting body formation (Liu et al., 2019a), pigment formation (Temp & Eggert, 1999), and flavonoid metabolism (Díaz et al., 2015).

Plant laccases, different from fungal laccases, are primarily involved in the process of lignin and anthocyanin biosynthesis. Earlier studies have indicated that many laccase genes are expressed in lignified tissues, and laccases could catalyze the oxidative polymerization of lignin precursors (Ranocha et al., 2002). Subsequent studies have further confirmed that laccase genes play important roles in lignin biosynthesis (Wang et al., 2015; Wang et al., 2008). For example, the cotton laccase gene overexpressing in poplar plants, could cause an increase in lignin content (Wang et al., 2008). On the contrary, overexpression of negative regulatory factors of laccase genes can lead to the decrease of lignin content in *Populus trichocarpa* (Lu et al., 2013). In addition, the various functions of laccase have also been reported in plants (Balasubramanian et al., 2016; Berthet et al., 2011; Bryan et al., 2016; Fang et al., 2015; Zhang et al., 2019). Laccase genes in *Arabidopsis* are also reported to express in interfascicular fibers and cell walls, and mediate the deposition of G lignin units in fibers and flavonoid polymerization, thus regulate tissue development (Berthet et al., 2011; Pourcel et al., 2005; Turlapati et al., 2011). Furthermore, laccase genes in sugarcane play important regulatory roles in catalyzing lignin biosynthesis, stress resistance, and morphogenesis, which indicate that laccase genes in sugarcane are involved in various pathways regulating plant growth (Igor et al., 2013; Zhang et al., 2019). It is worth mentioning that laccase genes are capable of responding to different abiotic and biotic stresses, according to various plant species. Many rice laccase genes have presented high expression levels in response to chilling, drought, salt and heavy metals (Cho et al., 2014; Liu et al., 2017). Among them, ectopic expression of rice laccase gene *OsChI1* (Os01g61160) in *Arabidopsis* could be associated with the production of some phenolic polymers, and enhanced flavonoid oxidation, thus lead to increased salt and drought tolerance (Cho et al., 2014). However, ectopic expression of *OsLAC10* could enhance tolerance of *Arabidopsis* to Cu stress, through lignification in roots that prevents excessive Cu from being absorbed (Liu et al., 2017). In other crops, laccase genes have also been reported on biotic and abiotic stresses (Hu et al., 2018; Le Roy et al., 2017). Subsequent studies have shown that, overexpression of the laccase gene *GhLac1* in cotton could also enhance defense response to both fungal pathogens and pests through increased lignification (Hu et al., 2018), *CsLAC* genes in citrus, activated by plant hormone SA, MeJA, and ABA, are predicted that these genes may function in citrus responding to pathogen infection (Xu et al., 2019). From the above results, laccase genes play an important role in the regulation of plant biotic and abiotic stresses.

Eggplant is an important vegetable crop with high nutritional value, in tropical and temperate regions. It often suffers from abiotic stresses, such as water deficit, high salinity, flooding and extreme temperature, and these stresses significantly reduce yield and even lead to complete loss of production (Wan et al., 2014). At the same time, these abiotic stresses can induce the expression of stress resistance genes in plants (Mwando et al., 2020). Considering that laccase genes play diverse roles in response to various stresses, an understanding of laccase functions may offer innovative approaches to enhancing the stress resistance of eggplant. Moreover, laccase genes have been further identified and characterized in Soybean (Wang et al., 2019), *Citrus sinensis* (Xu et al., 2019), *Sugarcane* (Zhang et al., 2019), *Setaria viridis* (Marcella Siqueira et al., 2020), *Eucalyptus* (Arcuri et al., 2020). However, no efforts have been taken to identify and characterize the laccase genes in eggplant. In this study, 48 laccase genes were identified from eggplant genome, and systematic analysis was executed in physical properties, phylogenetic evolution, chromosomal location, putative cis-regulatory element within promoters, conserved domains, gene structure and collinearity. Furthermore, the expression patterns of *SmLACs* were determined, in vegetative and reproductive organs at different developmental stages and also in response to abiotic stress. Taken together, our results would allow a better understanding of biological and physiological functions of laccase members in eggplant.

## Materials and Methods

### Identification of laccase genes in the eggplant genome

To identify members of the *SmLAC* gene family in eggplant, the genome assembly and annotation profile of *S.melongena* 67/3 line (Version 3.0) were downloaded from Sol Genomics Network (SGN, http://solgenomics.net/). A total of 17 laccase sequences from A*rabidopsis* were obtained according to published research (Turlapati et al., 2011). The reference sequences were used to search predicted proteins in eggplant based on genome files with an E-value cutoff of 1E−5. The candidate SmLAC proteins (SmLACs) were obtained after removing redundant and duplicate protein sequences. To further demonstrate the reliability of the candidate SmLAC proteins, a BLASTP search of the Swiss-Prot databases, was implemented, then all the putative proteins were confirmed by the Pfam (http://pfam.xfam.org/), NCBI CD-search (https://www.ncbi.nlm.nih.gov/Structure/cdd/wrpsb.cgi) and SMART database (http://smart.embl-heidelberg.de/). Finally, laccase members in eggplant with Cu-oxidase, Cu-oxidase_2, and Cu-oxidase_3 domains (PF00394, PF07731, and PF07732) were identified.

The annotation profile (Version 3.0) of eggplant *SmLAC*s localization on chromosomes was obtained from Sol Genomics Network (https://solgenomics.net/). TBtools was used to map the identified *SmLACs* to the chromosomes based on the annotation information and gene density of the eggplant genome. In order to better identify biological function of SmLACs in eggplant, the physicochemical properties of SmLACs, including MW (molecular weight), pI (theoretical isoelectric point), instability index, aliphatic index and grand average of hydropathicity, were analyzed by online software ExPASy (https://www.expasy.org). CELLO (v. 2.5, subcellular localization predictor, http://cello.life.nctu.edu.tw) was used to predict the subcellular localization of *SmLACs*.

### Phylogenetic analysis of SmLACs

In order to further clarify the classification, evolution and function of laccase members between eggplant and *Arabidopsis*, multiple sequence alignments were performed between SmLACs and 17 AtLACs from *Arabidopsis* containing typical characteristics using MEGA-X with the default parameters. An unrooted phylogenetic tree was constructed using the maximum-likelihood (ML) method with the following parameters: poisson correction, partial deletion, and 1,000 bootstrap replicates.

### Gene structure, conserved motifs and conserved domains analysis of SmLACs

In order to better understand relationship between *SmLACs* function and evolution, conserved motifs, conserved domains and gene structure of eggplant laccase members were analyzed. In order to identify conserved motifs, among all the *SmLAC*s, their protein sequences were subjected to online software MEME (http://meme-suite.org/tools/meme) using default parameters, except that the number of motifs was set to 10. Then, conserved domains were searched with SMART (http://smart.embl-heidelberg.de/#). All annotation information of these *SmLACs* was retrieved from the sol genomics network. Finally, conserved motifs, conserved domains and gene structure of eggplant laccase members were displayed using gene structure viewer in TBtools (Chen et al., 2020).

### Cis-elements, gene replication and synteny analysis of *SmLACs*

For identifying the cis-elements at the promoter regions of *SmLAC* genes, the 2.0 kb upstream sequence of the start codon (ATG) of each *SmLAC* gene was extracted from the eggplant genome sequence. A search was performed by using the PlantCARE server (http://bioinformatics.psb.ugent.be/webtools/plantcare/html/). Simultaneously, gene duplications of *SmLACs* were analyzed and illustrated with TBtools (Chen et al., 2020). Next, according to calculation method reported in a previous study (Liu et al., 2019b), the values of nonsynonymous (Ka) and synonymous (Ks) substitution rates of duplicated genes were calculated. MCScanX in TBtools (Chen et al., 2020) was later used to detect the synteny of *SmLAC* genes between eggplant and *Arabidopsis*, eggplant and tomato, with other parameters (filter collinearity in up genome, filter collinearity in down genome, filter gene in small collinearity in block:30).

### Expression analysis of *SmLACs* in different tissues

On the basis of RNA-seq data with SRA accession number SRP078398 reported in a previous study (Barchi et al., 2019), Expression values (FPKM) of *SmLACs* from 18 different tissues were extracted. It included seven different developmental stages of fruits, four different vegetative tissues, two different reproductive tissues, two different embryo tissues, two different developmental stages of buds and verticillium-inoc. roots 6 hpi. Finally, the expression values of laccase genes were transformed from log_2_ (FPKM+1), then the gene-wise normalized and hierarchically clustered heatmap was generated by TBtools software (Chen et al., 2020).

### Plant materials, growth conditions and cold stress

Plant materials used in this study were eggplant (*Solanum melongena* L.) variety Sanyueqie. Seedlings of eggplant were grown in pots containing organic substrate and vermiculite (3:1) mixtures, and then cultured at 25 °C under a photoperiod of 16 h (day) and 8 h (night) in a phytotron for two months. Then all the seedlings were treated at 4 °C for 12 h in a low temperature-programmable incubator, the second and third fully expanded leaves from the top were collected, immediately frozen in liquid nitrogen and then stored at −80 °C until they were used RNA extraction.

### RNA extraction, RNA-seq and quantification of gene expression levels under cold stress

The expression of the corresponding genes was also investigated using unpublished RNA-Seq datasets (Sequence Read Archive accessions SRR15036047, SRR15036048, SRR15036049 and SRR15036050). Specifically, total RNA was extracted with Invitrogen TRIzol® Reagent, then the transcriptome libraries were sequenced on the Illumina technology sequencing platform using the paired-end sequencing method (Novogene Co., Ltd, Tianjin, China). Two independent biological replicates for each treatment were performed for the transcriptome sequencing. Based on the subsequent published reference genome sequence from eggplant (Barchi et al., 2019), we used the TBtools (Chen et al., 2020) software to complete the analysis from the raw data to the gene expression level. In RNA-seq analysis, the gene expression levels can be estimated by counting the sequencing sequences (reads) located in genomic regions or gene exon regions. In addition to being directly proportional to the gene expression levels, the reads counts are also positively related to the length of the gene and the sequencing depth. In order to make the estimated gene expression levels of different genes and different experiments comparable, fragments per kb (kilo-base) per million fragments (FPKM) considers the effect of sequencing depth and gene length on fragment counts and is currently the most commonly used method for quantifying expression level of genes (Trapnell et al., 2010). The expression values of laccase genes were transformed from log_2_ (FPKM+1), then the gene-wise normalized and a hierarchically clustered heatmap was generated by TBtools software (Chen et al., 2020).

### Plant materials, growth conditions and stress treatments

In this study, we used eggplant variety “Sanyueqie” for plant materials. Seedlings of eggplant were grown in pots containing organic substrate and vermiculite (3:1) mixtures, and then cultured at 25 °C under a photoperiod of 16 h (day) and 8 h (night) in a phytotron for two months. Then all the seedlings were treated according to the following methods: (1) for cold stress, the plants were maintained at 4 °C in a low temperature-programmable incubator for 0, 1, 6, 12, 24 and 36 h; (2) for drought stress, the plants were irrigated with 20% PEG (cultured at 25 °C under a 16 h light/8 h dark cycle) for 0, 1, 6, 12, 24 and 36 h; and (3) for salinity stress, the plants were dipped in 200 mM NaCl and maintained at 25 °C with a 16 h light/8 h dark cycle for 0, 1, 6, 12, 24 and 36 h. At different time points, we collected the second and third fully expanded leaves from the top, immediately put them in liquid nitrogen, and then stored these samples at −80 °C for RNA extraction. In all abiotic stress treatments, we performed three biological replications independently.

### RNA extraction, and expression analysis

We extracted total RNA from leaves of treated and control group eggplant seedlings using RNAprep Pure Plant Kit (TIANGEN) according to the manufacturer’s specifications, and then performed quantification with a two-step reaction process: reverse transcription (RT) and qPCR. Reverse transcription was performed in 2,720 Thermal Cycler (Applied Biosystems™). qPCR reaction was performed on a CFX96 Real-Time PCR (Bio-Rad, Hercules, CA, USA). At the end of the PCR cycles, we performed analysis of the melting curve to validate the specific generation of the expected PCR product. Adenine phosphoribosyl transferase (APRT, accession JX448345, (Gantasala et al., 2013)) from eggplant was used as the internal control. The relevant primers are listed in Table S1. All reactions were run in triplicate. The relative expression values of *SmLAC* genes were calculated using the 2^−ΔΔCt^ method.

### Statistical analysis

Data analyses were conducted using the SPSS version 25.0 (SPSS, Chicago, IL, USA) statistical software. For all analysis, the level of significance was set at *p* < 0.05. Column charts were drawn using Excel 2019 software. Sample variability was given as standard deviation (SD).

## Results

### Identification of laccase gene members in eggplant genome

**Figure 1:**
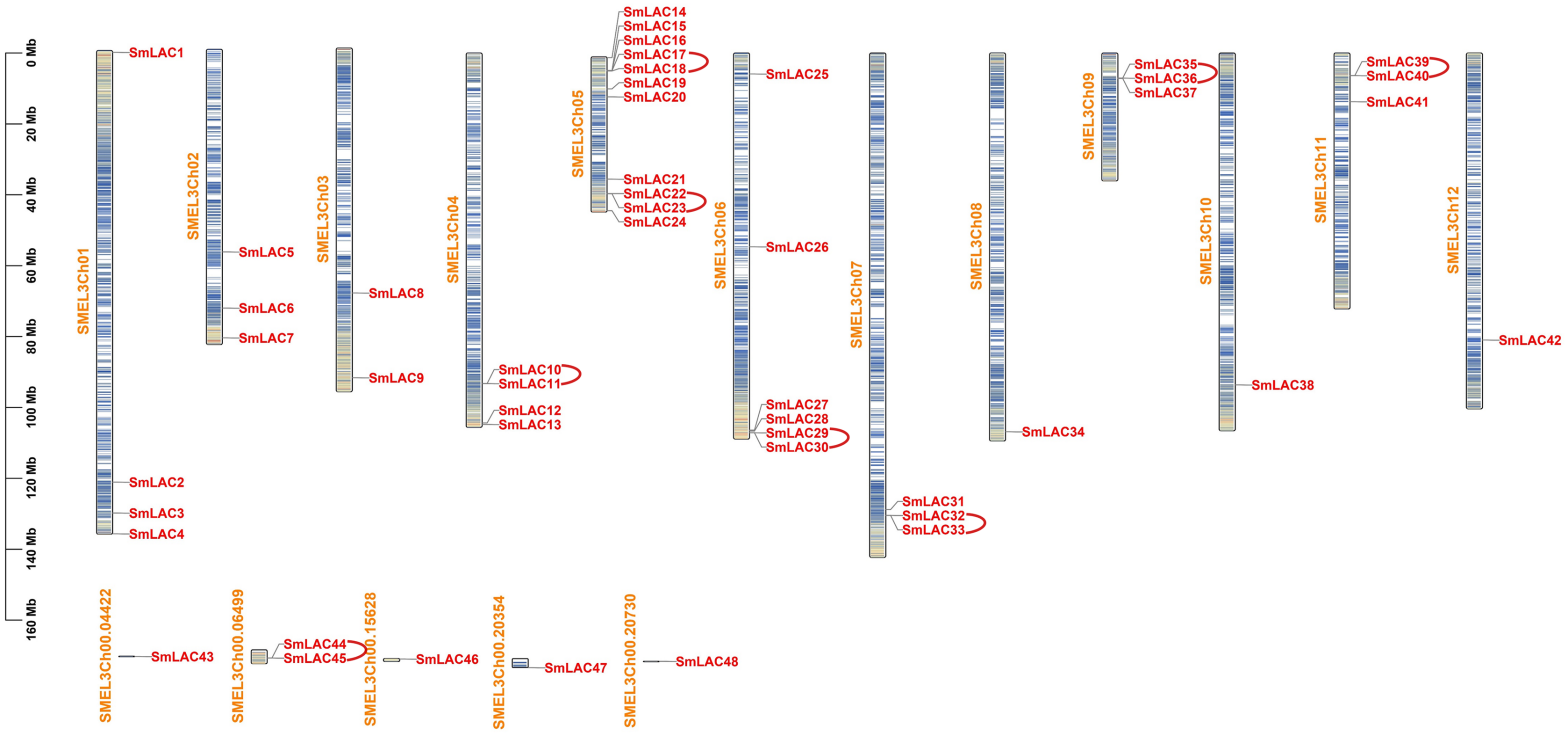
Chromosomal distributions and tandem replication analysis of *SmLACs* in eggplant. Scale bar on the left indicates chromosome lengths (Mb). Columns with different colors denote gene density on each chromosome. Red represents the distribution of different *SmLACs* on each chromosome, yellow represents the number of each chromosome, and arcs in red indicate tandem replication genes existing in laccase members from eggplant.

To identify the laccase family members in eggplant, we downloaded the genome assembly and annotation profile of eggplant (Version 3.0) from Sol Genomics Network. Subsequently, seventeen laccase sequences from A*rabidopsis* as reference sequences were used to search predicted proteins in eggplant based on genome files with an E-value cutoff of 1E−5. After removing redundant peptide sequences with only one or two typical domains, 48 laccase family members in eggplant were identified, for specific coding region sequences and peptide sequences, please see Tables S2 and S3. These members were then numbered according to their location on the chromosomes and designated *SmLAC1* through *SmLAC48* (Table S4). Moreover, 42 *SmLAC* gene members were widely distributed on 12 chromosomes and six *SmLAC* genes were associated with scaffolds (Fig. 1). Notably, chromosome 5 contained the largest number of laccase genes, 11 genes (*SmLAC14*-*SmLAC24*) were spread out along chromosome 5. On the contrary, chromosomes 8, 10 and 12 contained only one gene respectively. In addition, six genes (*SmLAC43*-*SmLAC48*) were not mapped onto any chromosome. Then physico-chemical properties of 48 SmLAC family members were further analyzed. The length of SmLAC proteins ranged from 494 to 835 amino acids (581 amino acids on average), with molecular weight ranging from 55.32 to 93.41 kDa (64.91 kDa on average), and isoelectric points (pI) varying from 5.38 to 9.60 (8.50 on average). In addition, most of SmLAC proteins instability index values were below 40.0, whereas instability index values of only four SmLAC proteins were above 40, which indicated that most of the eggplant laccase proteins belonged to stable proteins. Furthermore, the grand average of hydropathicity of all eggplant laccase proteins was below 0.0, it implied that all eggplant laccase proteins were hydrophilic proteins.

### Analysis of the phylogeny of eggplant laccase family

**Figure 2:**
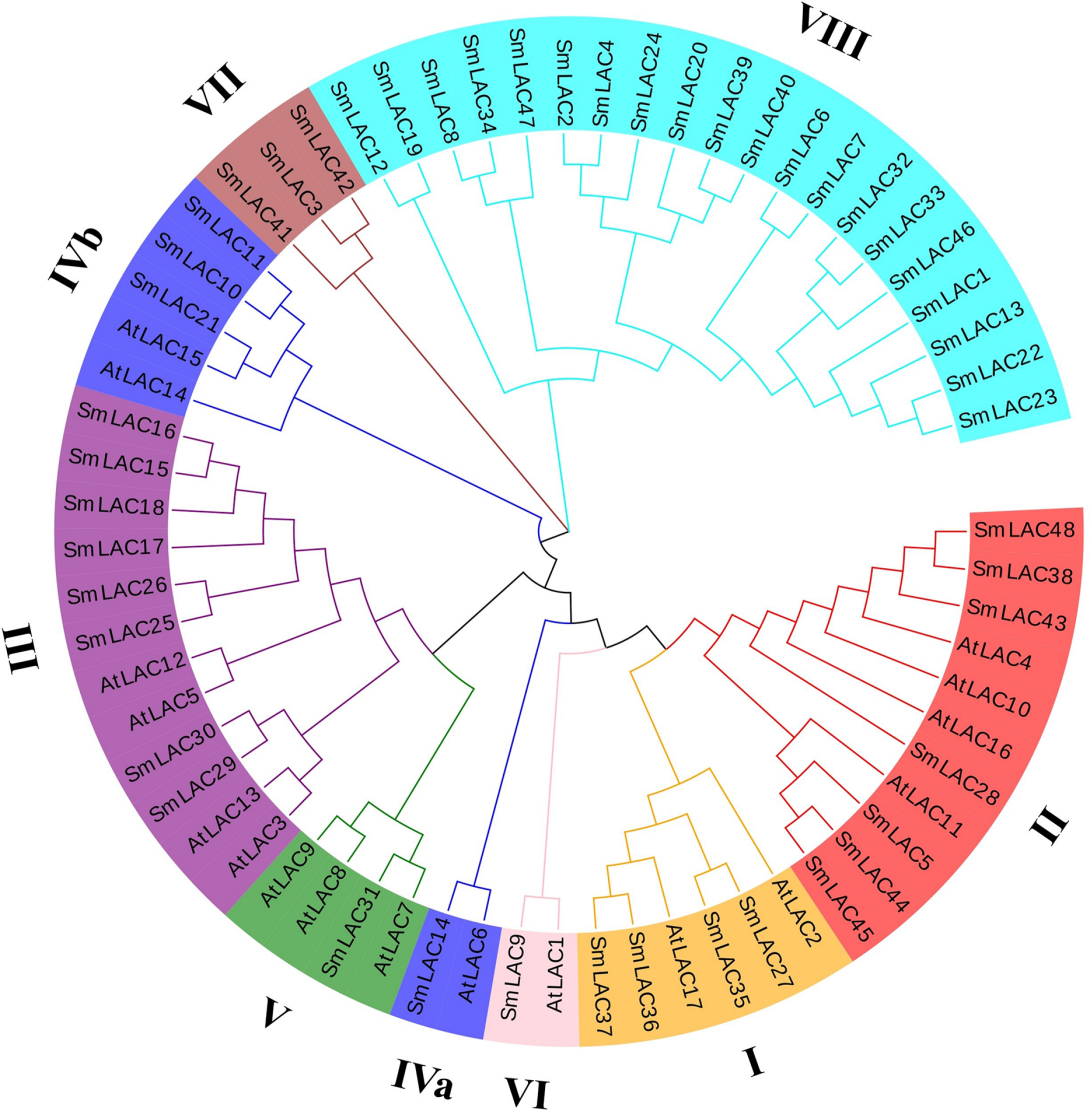
Phylogenetic analysis of laccase family members from eggplant and *Arabidopsis*. A total of 48 SmLACs and 17 AtLACs containing typical characteristics from *Arabidopsis* were used to construct the unrooted phylogenetic tree, using MEGA-X by the maximum-likelihood (ML) method with 1,000 bootstrap replicates, based on the full-length peptide sequences of LACs. The SmLAC proteins were divided into eight different groups.

To further study the phylogenetic relationships of laccases between eggplant and *Arabidopsis*, an unrooted phylogenetic tree was constructed using the maximum-likelihood (ML) method. On the basis of six different groups previously reported for *Arabidopsis*, 25 eggplant laccase members were divided into six groups, but the laccase members from eggplant were not equally distributed in each group, eight family members were observed belonging to group III, seven members belonging to group II, four members belonging to group I, and one member each belonging to group V and group VI (Fig. 2). Moreover, eggplant laccase members in group IV were classified into two subgroups: subgroup IVa (SmLAC14) and subgroup IVb (SmLAC10, SmLAC11 and SmLAC21). In addition, the remaining 23 SmLAC members found no hits among the AtLACs, they were temporarily classified as group VII and group VIII (Fig. 2).

### Analysis of gene structure, motif compositions and conserved domains of eggplant laccase family members

**Figure 3:**
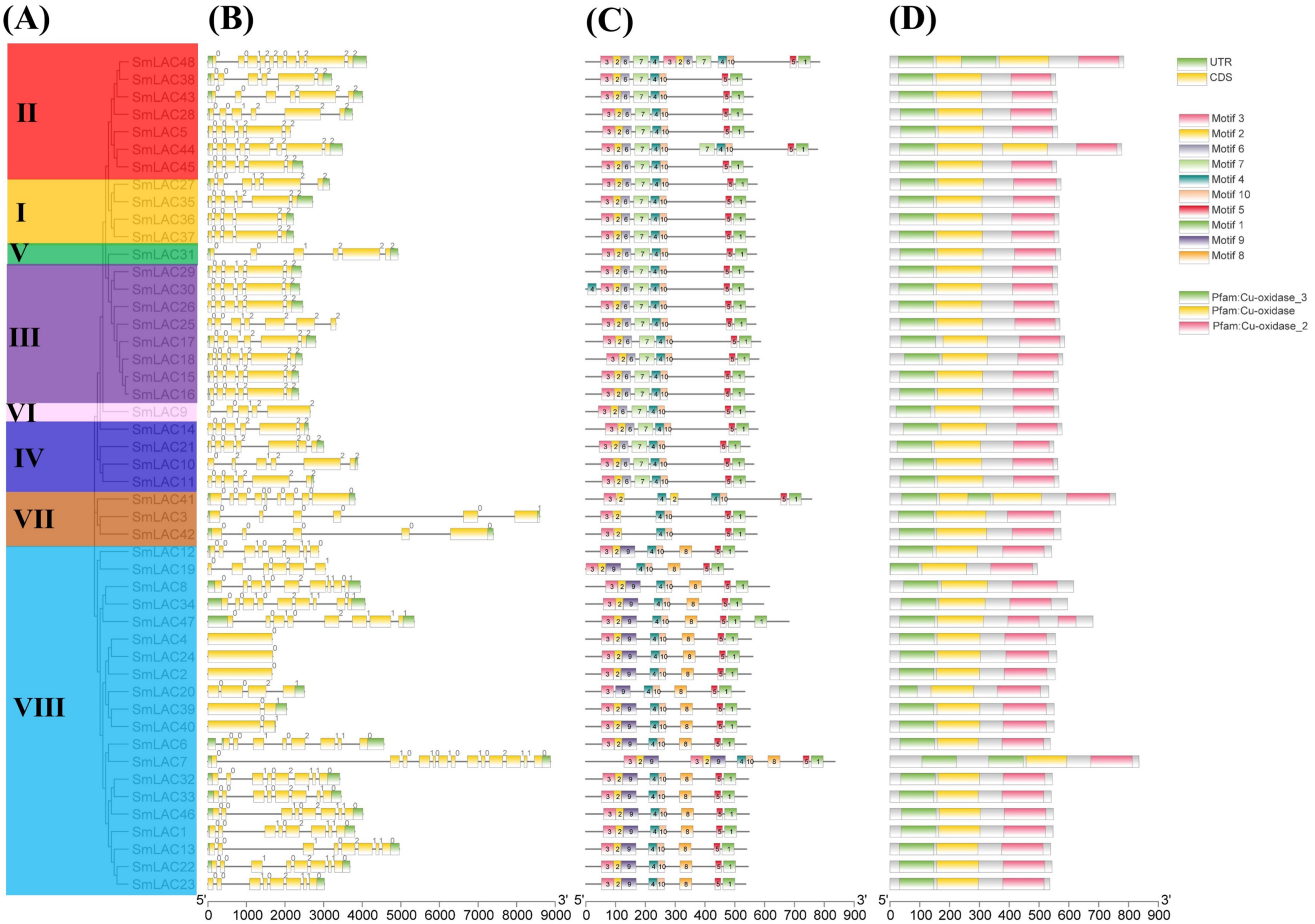
Phylogenetic relationships, motifs and gene structures of laccase members from eggplant. (A) The phylogenetic tree was constructed using the maximum-likelihood (ML) method with 1,000 replications, based on the multiple alignments of 48 laccase amino acid sequences from eggplant. The eight major groups were indicated and were differentiated with diverse colored backgrounds. (B) Gene structures of *LAC* genes from eggplant. CDS, UTR and introns were shown by green boxes, yellow boxes and black lines, respectively. (C) Schematic of the conserved motifs in the LAC proteins from eggplant elucidated by TBtools. Every amino acid motif in the SmLAC proteins (1–10) was represented by one colored box with a number. The black lines indicated relative protein lengths. Conserved motifs were defined by MEME. (D) Schematic of the conserved domains in the LAC proteins from eggplant elucidated by TBtools. The three conserved domains including Cu-oxidase, Cu-oxidase_2 and Cu-oxidase_3 were shown by yellow boxes, pink boxes and green lines, respectively. Conserved domains were defined by SMART.

To show the structural diversity of eggplant laccase family members, exon/intron organizations, conserved motifs and domains were analyzed based on the phylogenetic tree of all eggplant laccase members alignments (Fig. 3). Our results showed that almost all the closest genes encoding those SmLACs on the phylogenetic tree showed remarkably analogous gene structures. Moreover, nearly 40% of *SmLAC* gene members included six exons, and the number of exons varied from 1 to 13 (0 to 12 introns) (Fig. 3). However, there was still a small proportion of the gene clades that exhibited different intron/exon organizations. For instance, *SmLAC44* had eight exons, whereas its nearby paralogous genes, *SmLAC5*, *SmLAC28*, *SmLAC38*, *SmLAC43* and *SmLAC45*, typically had six exons even though their evolutionary relationships reached 87–100% bootstrap values, respectively. Conserved motifs of the 48 SmLACs were analyzed through the MEME program. It was discovered that six conserved motifs including motif 1, motif 2, motif 3, motif 4, motif 5 and motif 10 were found in all 48 putative SmLAC members. Moreover, the motif compositions in the same group possessed similar structures and organizations. SmLACs from group I, group II (except for SmLAC44 and SmLAC48), group III (except for SmLAC30), group IV, group V and group VI contained completely the same motif composition, which suggested the possibility of functional redundancies among these members (Fig. 3). The conserved motifs might play critical roles in mediating functions of SmLACs. Furthermore, the 48 eggplant laccase members contained three conserved domains of Cu-oxidase, Cu-oxidase_2 and Cu-oxidase_3 identified by SMART (Fig. 3).

### Analysis of the cis-regulatory elements predicted in the promoters of *SmLACs*

To find possible regulatory patterns of the *SmLACs*, we extracted the 2.0 kb upstream sequence of the start codon (ATG) of each *SmLAC* gene (Table S5), and examined the putative cis-regulatory elements in the promoter regions of eggplant laccase genes by PlantCARE. All *SmLAC* promoter regions were predicted to contain many light responsive elements (Table 1), such as G-box, GT1-motif and so on, which indicated important roles in eggplant morphogenesis. In addition, abscisic acid (ABA) and anaerobic induction (AAI) relative elements were more frequently identified in the promoter regions of laccases (Table 1). Simultaneously, other important representative cis-regulatory elements were also predicted. Among them, in the promoters of the *SmLACs*, a great number of cis-elements associated with hormone response were identified in many laccase genes (Table 1). For example, 39 out of 48 laccases included ABA-responsive cis-regulatory elements, 27 laccases contained gibberellins(GA) response elements, 26 laccases had salicylic acid (SA) responsive elements, and 21 laccases contained cis-regulatory elements in relation with auxin and methyl jasmonate (Me-JA) respectively (Table 1).

**Table 1:** The putative cis-regulatory elements in the promoter regions of eggplant laccase genes.

Promoter	LR	CC	FB	Phytohormone response					Stress response					Tissue			
				ABA	AUX	GA	Me-JA	SA	AAI	DS	Dt	LT	Wound	Meristem	Endosperm	Seed	Zein metabolism
SmLAC1	Y	Y	N	Y	N	Y	Y	Y	N	Y	Y	N	N	Y	N	N	N
SmLAC2	Y	N	N	Y	N	Y	Y	N	N	Y	N	Y	N	N	Y	N	N
SmLAC3	Y	N	N	Y	N	N	Y	Y	N	N	N	N	N	N	N	N	N
SmLAC4	Y	N	N	Y	N	Y	Y	Y	Y	Y	Y	N	N	Y	N	N	N
SmLAC5	Y	N	N	Y	N	Y	N	Y	Y	Y	N	N	N	N	Y	N	Y
SmLAC6	Y	Y	N	N	N	Y	N	Y	Y	N	N	Y	N	Y	N	N	N
SmLAC7	Y	N	N	Y	N	N	N	N	Y	Y	N	N	N	N	N	N	N
SmLAC8	Y	N	N	N	N	N	N	Y	Y	Y	Y	Y	N	N	N	N	N
SmLAC9	Y	Y	N	Y	Y	N	Y	N	Y	N	Y	N	N	N	N	N	N
SmLAC10	Y	N	N	N	Y	N	Y	N	Y	Y	N	N	N	N	Y	N	Y
SmLAC11	Y	N	N	Y	Y	N	Y	Y	Y	N	N	N	Y	N	Y	N	Y
SmLAC12	Y	N	N	Y	N	Y	N	Y	Y	N	Y	N	N	N	N	N	Y
SmLAC13	Y	N	N	N	N	Y	N	Y	Y	Y	Y	N	N	N	N	N	N
SmLAC14	Y	N	N	Y	Y	Y	Y	N	Y	Y	N	N	N	N	N	Y	Y
SmLAC15	Y	N	N	Y	N	N	Y	Y	Y	Y	N	Y	N	Y	Y	N	N
SmLAC16	Y	N	N	Y	N	N	Y	Y	N	Y	N	N	N	Y	Y	N	N
SmLAC17	Y	N	N	Y	Y	Y	N	N	Y	Y	Y	N	N	N	N	N	N
SmLAC18	Y	Y	N	N	N	N	N	Y	N	Y	N	Y	N	Y	N	N	Y
SmLAC19	Y	N	Y	Y	Y	Y	Y	N	Y	N	N	Y	N	Y	Y	N	N
SmLAC20	Y	N	N	Y	N	Y	Y	Y	Y	N	N	Y	N	N	N	N	N
SmLAC21	Y	N	N	Y	N	Y	N	N	Y	N	N	N	N	N	N	N	N
SmLAC22	Y	Y	N	Y	Y	N	N	N	Y	N	Y	N	N	Y	Y	N	N
SmLAC23	Y	N	N	Y	Y	Y	N	Y	Y	N	N	Y	N	N	N	N	N
SmLAC24	Y	N	N	Y	Y	Y	Y	Y	Y	N	N	N	N	Y	N	N	Y
SmLAC25	Y	N	N	Y	N	N	N	Y	Y	N	N	Y	N	N	Y	N	Y
SmLAC26	Y	N	N	Y	N	Y	N	N	Y	N	Y	N	N	N	N	N	N
SmLAC27	Y	Y	N	N	Y	N	N	N	Y	Y	N	N	N	Y	N	N	N
SmLAC28	Y	Y	N	Y	Y	N	Y	Y	Y	Y	N	N	N	N	N	N	Y
SmLAC29	Y	N	N	Y	N	Y	N	Y	Y	Y	Y	N	Y	N	N	N	Y
SmLAC30	Y	N	N	N	N	Y	Y	N	Y	N	Y	Y	N	N	N	N	N
SmLAC31	Y	N	N	Y	Y	Y	N	N	Y	Y	N	N	N	Y	N	N	N
SmLAC32	Y	N	Y	Y	Y	Y	N	N	Y	N	N	N	N	Y	N	N	N
SmLAC33	Y	N	N	Y	N	N	N	Y	Y	Y	Y	N	N	N	N	N	Y
SmLAC34	Y	N	N	N	Y	Y	N	Y	N	N	Y	N	N	Y	N	N	Y
SmLAC35	Y	Y	Y	Y	Y	N	Y	Y	Y	N	N	Y	N	Y	N	N	Y
SmLAC36	Y	N	N	Y	N	N	N	N	N	N	Y	N	N	N	Y	N	N
SmLAC37	Y	N	N	Y	Y	Y	Y	N	N	Y	Y	N	N	N	Y	N	Y
SmLAC38	Y	Y	N	N	Y	Y	N	N	Y	N	Y	N	N	N	Y	N	Y
SmLAC39	Y	Y	N	Y	Y	Y	Y	Y	Y	N	Y	N	N	N	N	N	N
SmLAC40	Y	N	Y	Y	Y	N	Y	N	Y	Y	Y	N	N	Y	N	N	N
SmLAC41	Y	Y	N	Y	N	Y	N	Y	N	Y	N	N	N	Y	N	N	N
SmLAC42	Y	N	N	Y	N	Y	N	Y	N	N	N	N	N	N	N	N	N
SmLAC43	Y	N	N	Y	N	Y	N	N	N	Y	N	N	N	N	N	N	N
SmLAC44	Y	N	N	Y	Y	Y	Y	N	Y	N	N	N	N	N	N	N	N
SmLAC45	Y	Y	N	Y	N	N	Y	N	N	N	N	N	N	N	Y	N	N
SmLAC46	Y	N	N	Y	N	N	N	Y	Y	Y	Y	N	N	N	N	N	N
SmLAC47	Y	N	N	Y	N	N	N	N	Y	N	N	N	N	N	N	N	N
SmLAC48	Y	N	Y	Y	Y	N	N	Y	N	N	Y	N	N	Y	N	N	N

**Note:**

LR, Light responsive; CC, Circadian control; FB, Flavonoid biosynthetic; ABA, Abscisic acid; AUX, Auxin; GA, Gibberellins; MJ, Methyl jasmonate; SA, Salicylic acid; AAI, Anaerobic induction; DS, Defense and stress; Dt, Drought; LT, Low temperature; Y represents the presence of regulatory elements; N represents the absence of regulatory elements.

In the same way, a great number of stress-responsive cis-elements, in relation with anaerobic induction, defense and stress, drought, low temperature and wound, were also detected in the promoters of the *SmLACs* (Table 1). For instance, stress-responsive cis-regulatory elements, responding to AAI, were detected most in the promoters of 35 *SmLACs*, while stress-responsive cis-elements, related to wound, were predicted least in the promoters of two *SmLACs*. At the same time, tissue specific cis-regulatory elements, related to the development of meristem, endosperm, seed and zein metabolism, were also predicted within the promoters of many laccase genes from eggplant (Table 1). Among them, cis-regulatory elements, related to development of meristem, were found most in the promoters of 17 *SmLACs*, but cis-regulatory elements, related to the development of seed, were discovered only in the promoter of *SmLAC14* (Table 1). Furthermore, MYB binding sites, involved in flavonoid biosynthetic genes regulation, were also found in the promoters of five *SmLACs*, including *SmLAC19*, *SmLAC32*, *SmLAC35*, *SmLAC40* and *SmLAC48*, which indicated that these laccases may be related to the process of flavonoid biosynthesis in eggplant.

### Analysis of gene duplication and collinearity in eggplant laccase family

Gene duplication, including tandem repeats, segmental repeats and interspersed repeats, is considered to be the main driving force under the process of genome evolution. Interestingly, we discovered that two pairs of eggplant laccase genes (*SmLAC15/SmLAC16* and *SmLAC36/SmLAC37*) contained the same protein coding sequences and physico-chemical properties, so they were temporarily identified as interspersed repeats. In order to gain insight into the eggplant laccase gene duplication pattern, we further analyzed the tandem and segmental duplication events of eggplant laccase genes by TBtools. In this study, we discovered that eight pairs of 48 laccase genes belonged to tandem repeats (Fig. 1, Table S6). However, there were no segmental repeats in the eggplant laccase gene family. Then we obtained Ka and Ks values of each duplicated *SmLAC* gene pair, using simple Ka/Ks calculator in Tbtools. It was discovered that the Ks values of tandem repeats were between 0.043 and 2.038. Moreover, 75% (6/8) of Ks values were less than 1. In addition, we calculated Ka/Ks values of all *SmLAC* gene pairs, and discovered that all the Ka/Ks values were less than 1, which revealed these genes had evolved in the selection of strong purification. Furthermore, we calculated the approximate dates of *SmLAC* gene duplication events, in accordance with the method reported in a previous study(Liu et al., 2019b). The duplication events of *SmLAC* genes happened from 8.27 Mya (Ks = 0.043) to 391.86 Mya (Ks = 2.04), with an average of 113.05 Mya (Ks = 0.59). Detailed information in regard to duplication gene pairs, duplication type, Ka, Ks, Ka/Ks and approximate duplication date (Mya) of all identified duplicated *SmLAC* genes were listed in Table S6.

**Figure 4:**
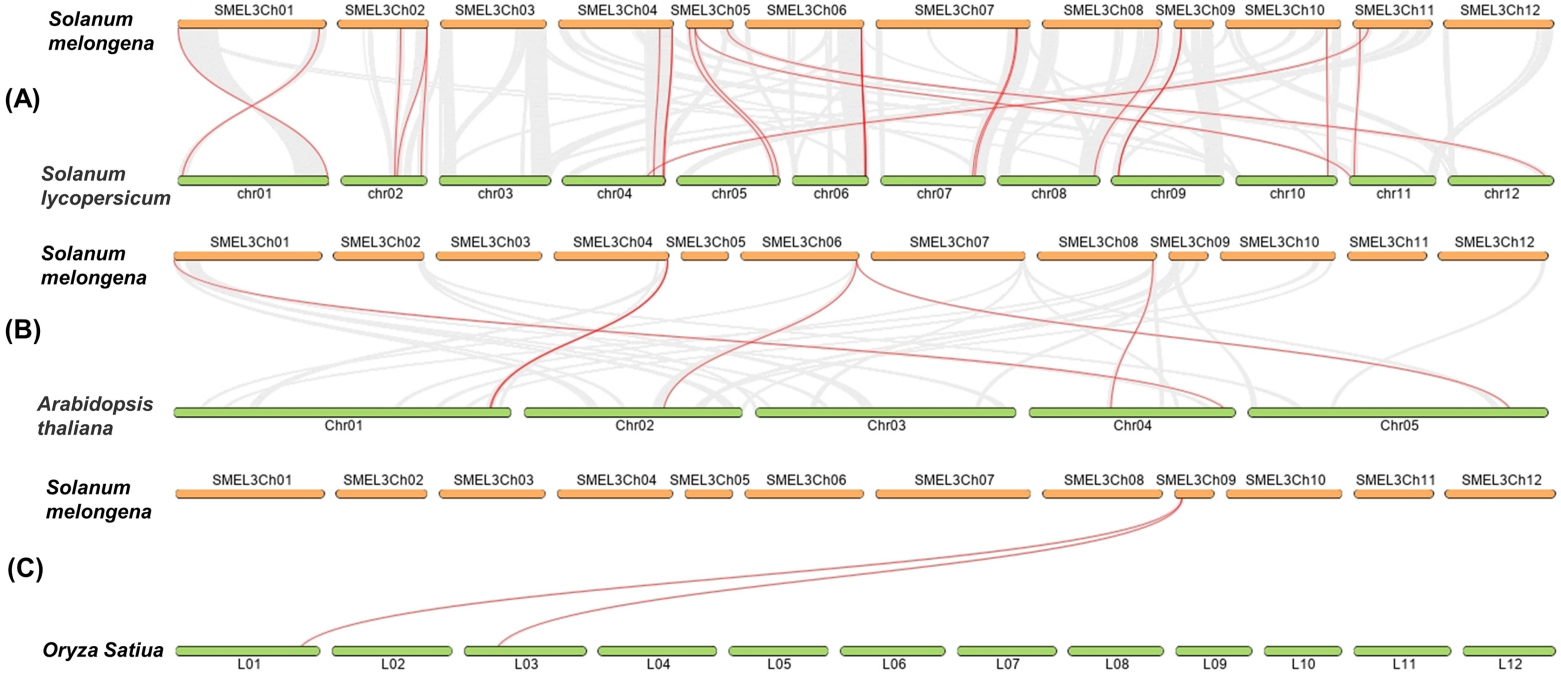
(A–C) Synteny analysis of *LAC* genes between eggplant and tomato, eggplant and *Arabidopsis*, eggplant and rice. The red lines represented collinearity relationship between eggplant and tomato, eggplant and *Arabidopsis*, eggplant and rice.

In order to detect the homology of eggplant laccase genes, we analyzed the collinearity, between eggplant and tomato, eggplant and *Arabidopsis thaliana*, eggplant and rice, with Dual Systeny Plot for MCscanX in TBtools. As shown in Fig. 4 and Table S7, there were twenty-four pairs of collinearity between twenty-one *SmLAC* genes and twenty-four *SlLAC* genes, six pairs of collinearity between six *SmLAC* genes and six *AtLAC* genes, but only two pairs of collinearity between one *SmLAC* gene and two *OsLAC* genes. Moreover, twenty-one *SmLAC* genes were collinear with *LAC* genes from *Arabidopsis*, tomato or rice. Unfortunately, none of *SmLAC* genes were collinear with *LAC* genes in other three species.

### Analysis of expression patterns of *SmLACs* in different tissues

**Figure 5:**
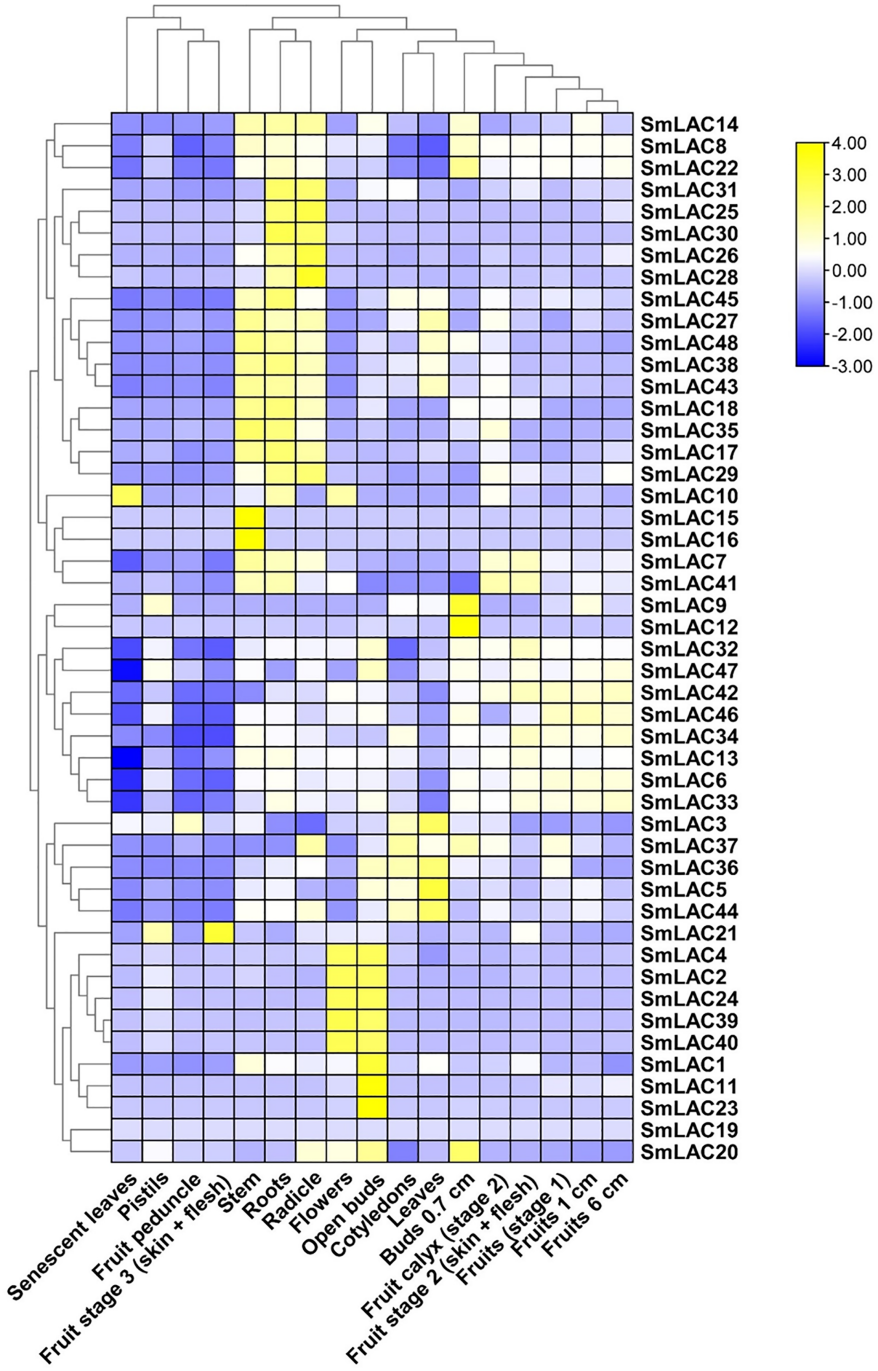
Heat map representation of SmLACs in various development stages. The names of the genes were written on the right of the heat map. The bar at the right of the heat map represented relative expression values, and the relative expression values were log_2_ (FPKM+1). So that the relatively low expression values or relatively high expression values were colored dark blue or yellow with increasing intensity, respectively. But no expression values were colored white.

To determine the function of *SmLAC* genes in eggplant in different developmental stages, we analyzed the expression patterns of forty-eight *SmLAC* genes in various vegetative and reproductive stages of eggplant, based on RNA-seq data reported in previous study (Barchi et al., 2019). Overall, according to the data, we classified the expression patterns of *SmLAC* genes into three types (no-expression, constitutive expression and specific expression) (Fig. 5 and Table S8). Among them, gene *SmLAC19* was not expressed in all of the examined developmental stages. By contrast, seventeen *SmLAC* genes were ubiquitously expressed in all eggplant organs/tissues, but differentially expressed in different tissues. Among them, *SmLAC6, SmLAC8, SmLAC34 and SmLAC47* genes showed high expression levels in all tissues/organs, which indicated that these genes may involve in metabolism, plant growth and development. But thirteen *SmLAC* genes were discovered at high level in many tissues/organs, but low FPKM values were found in some organs. For instance, gene *SmLAC13* was accumulated at a relatively higher level in roots, fruits and flowers, yet at the lowest level in senescent leaves. In addition, the other thirty-one *SmLAC* genes showed relatively high level in some organs, but a relatively low level or no-expression in many tissues/organs. For example, *SmLAC2*, *SmLAC24*, *SmLAC39* and *SmLAC40* were detected at relatively high levels in flowers and open buds, while at a lower to no-expression level in other tissues (Fig. 5 and Table S8).

### Expression analysis of *SmLAC* genes under cold stress

**Figure 6:**
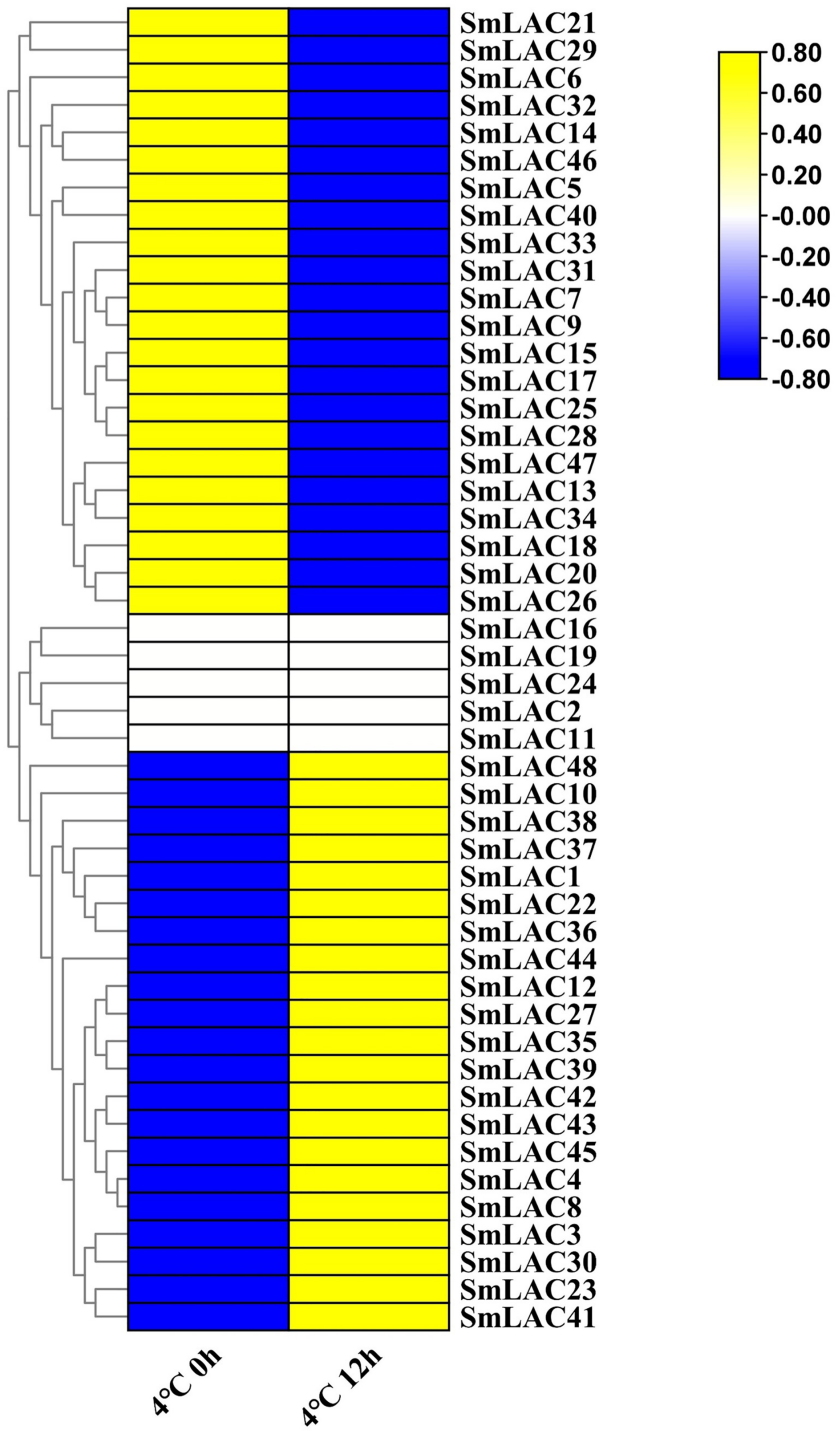
Heat map representation of *SmLACs* under cold stress. The names of the genes were written on the right of the heat map. The bar at the right of the heat map represented relative expression values, and the relative expression values were log_2_ (FPKM+1). So that the relatively low expression values or relatively high expression values were colored dark blue or yellow with increasing intensity, respectively. But no expression values were colored white.

According to our unpublished RNA-seq data under cold stress, a heat map was displayed on the basis of log_2_ transformed (FPKM+1) values. Compared with normal growth conditions, twenty-one *SmLAC* genes were up-regulated, which indicated that 43.75% of laccase members were induced by cold stress (Fig. 6 and Table S9). On the contrary, twenty-two *SmLAC* genes were down-regulated under cold stress for 12 h, which suggested that 43.75% of laccase members exhibited suppressed expression. In addition, five *SmLAC* genes (*SmLAC2*, *SmLAC11*, *SmLAC16*, *SmLAC19* and *SmLAC24*) were detected with no expression values in both conditions, which was basically consistent with the above published RNA-seq data regarding the expression of the laccase family gene in the leaf tissue. Unfortunately, no significant differentially expressed genes have been found based on RNA-seq data under cold stress. From this result, it was suggested that cold stress could not significantly affect the expression of laccase family genes.

### Expression patterns of *SmLAC* genes in response to cold, drought and salt tress

**Figure 7:**
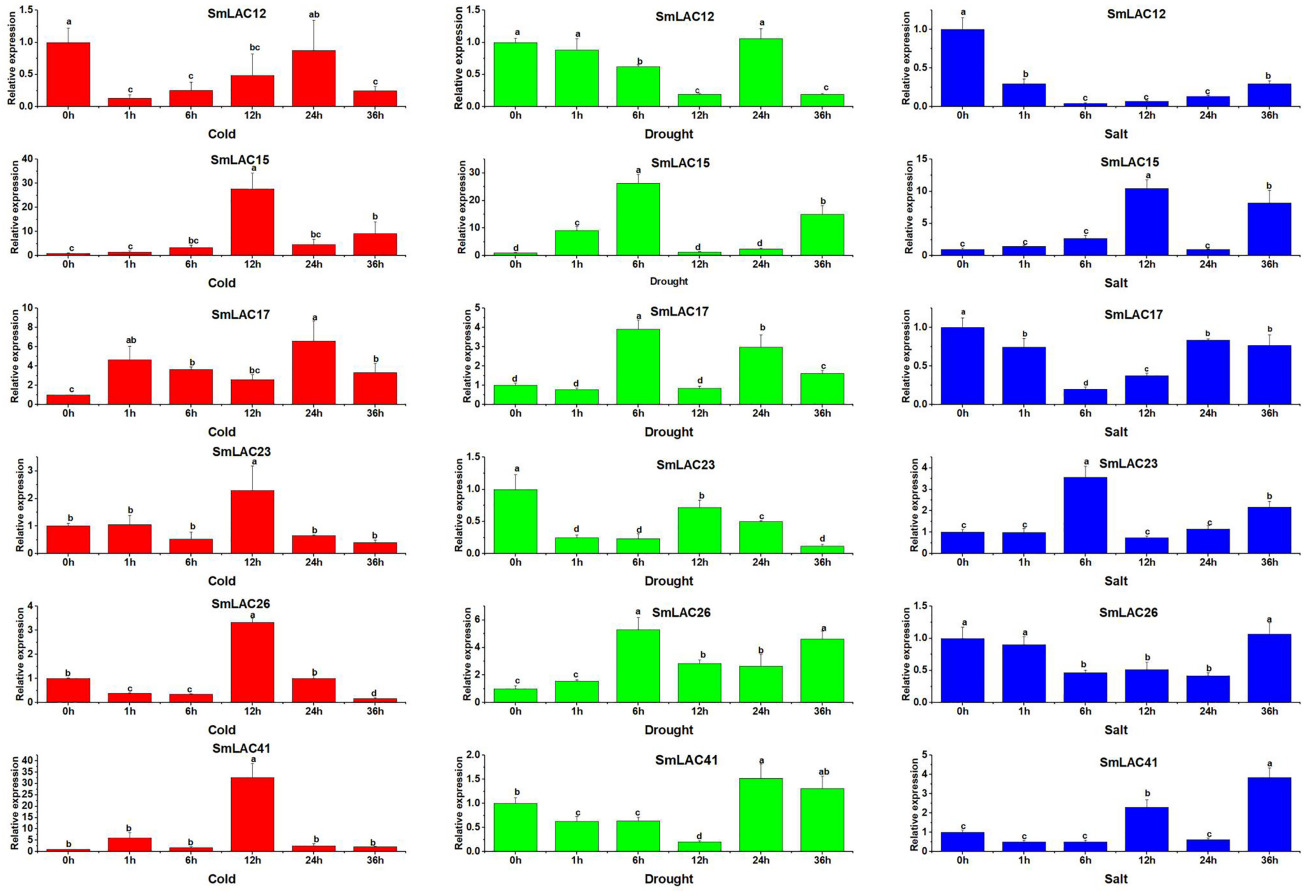
Expression analysis of six *SmLAC* genes selected under cold, drought and salt stress. Values were means of three replicates ± standard deviation (SD). Different letters indicated significant differences (ANOVA analysis, *P* < 0.05).

On the basis of the potential cis-regulatory elements of *SmLACs* promoters, it was indicated that *SmLAC* genes may respond to various stresses. Therefore, six *SmLAC* genes (*SmLAC12*, *SmLAC15*, *SmLAC17*, *SmLAC23* and *SmLAC41*) were selected to detect their expression patterns under various stress conditions. As shown in Fig. 7, for cold stress, the expression levels of *SmLAC15*, *SmLAC17*, *SmLAC23* and *SmLAC41* were significantly up-regulated on the whole. Among them, the expression levels of *SmLAC15*, *SmLAC23* and *SmLAC41* were upregulated most significantly at 12 h (27.7, 2.3 and 32.8 folds as much as that of the control respectively), while the relative expression value of gene *SmLAC17* showed the most significant level at 24 h. On the contrary, gene *SmLAC12* was significantly down-regulated at different time points, except for 24 h. Whereas, gene *SmLAC26* showed significantly down-regulated expression at 1, 6, and 36 h, but increased sharply to a significant up-regulated expression level at 12 h. Under drought treatment, the expression values of *SmLAC15, SmLAC17* and *SmLAC26* exhibited up-regulated change within 6 h, and showed peak expression at 6 h, then declined. With the increase of processing time, *SmLAC26* was still significantly up-regulated at 12, 24 and 36 h, but *SmLAC17* reached significantly up-regulated expression at 24 and 36 h again, and *SmLAC15* only at the time point of 36 h. In addition, the expression values of *SmLAC12*, *SmLAC23* and *SmLAC41* exhibited similar change tendencies (first declined, increased and then declined). Overall, compared with the control, gene *SmLAC23* emerged significantly down-regulated levels at different time points, but *SmLAC12* and *SmLAC41* possessed relatively complex expression changes, first displayed continuously down-regulated expression within 12 h, then *SmLAC12* attained a significant down-regulation level again at 36 h, while *SmLAC41* showed a significant up-regulation level at 24 h. As for salt treatment, both of *SmLAC12* and *SmLAC17* displayed significantly down-regulated levels at different time points, but *SmLAC26* showed significantly inhibited expression level only at 6, 12 and 24 h. On the contrary, *SmLAC15* and *SmLAC41* acquired significantly induced expression levels at 12 and 36 h, while *SmLAC23* obtained significantly induced expression levels at 6 and 36 h, but none of the three genes reached the levels of significant difference at other time points. From the above results, we can learn that the selected *SmLAC* genes except for *SmLAC12* were significantly induced by one or multiple stresses.

## Discussion

Laccases belong to multicopper oxidases encoded by a multigene family, and play crucial roles in flavonoid biosynthesis and oxidation of monolignols to produce higher-order lignin involved in plant development and stress responses. In this study, 48 laccase genes, including three copper oxidase domains, were identified based on genome assembly and annotation profile of eggplant (Table S4), which is considerably higher than those previously reported in other crops (Liu et al., 2017; Wang et al., 2017a; Xu et al., 2019; Zhang et al., 2019), but slightly lower than this recently reported in *Eucalyptus grandis* (Arcuri et al., 2020). Chromosomal localization analysis indicates that most *SmLAC* genes are widely distributed in 12 chromosomes (Fig. 1). Similar results have been discovered in the chromosomal location of laccase genes in soybean, due to their presence on unanchored scaffolds (Wang et al., 2019).

Further analysis of many predicted physico-chemical properties has showed that predicted isoelectric points (pI) of forty-three laccases from eggplant were higher than 7, implying that they are more accustomed to function in an alkaline environment (Table S4). Similar result is also reported in soybean (Wang et al., 2019). Moreover, it was observed that the great majority of eggplant laccases belong to secretory proteins, as indicated by the presence of an N-terminal signal peptide (Table S4). This result is consistent with the research of soybean laccases (Wang et al., 2019).

On the basis of six different groups previously reported for *Arabidopsis* (Turlapati et al., 2011), 25 eggplant laccase members were divided into six groups, through multiple sequence alignments of laccase members between *Arabidopsis* and eggplant, but the members from eggplant were not equally distributed in each group (Fig. 2). For instance, SmLAC31 was clustered with AtLAC7 induced by iron deficiency, which is involved in abiotic stress (Turlapati et al., 2011). Moreover, promoter regions of gene *SmLAC31* were predicted to include cis-regulatory elements of phytohormone and stress response (Table 1). Furthermore, eggplant laccase members from Group I and Group II were classified with AtLAC4 and AtLAC17 related to lignin biosynthesis (Berthet et al., 2011; Zhao et al., 2013), implying that the eggplant laccases seem to involve in lignin biosynthesis. In addition, like in soybean, it was worth noting that Group IV was divided into two different branches, IVa and IVb. Group IVa included eggplant laccase SmLAC14 and *Arabidopsis* laccase AtLAC6, reported to be inhibited by cold and osmotic stress (Turlapati et al., 2011), predicting that SmLAC14 may have similar functions to AtLAC6. Further results showed that the promoter region of *SmLAC14* was detected to include many cis-regulatory elements involved in hormonal response, but no cis-regulatory elements in response to cold or osmotic stress (Table 1), and *SmLAC14* was discovered to show high expression values in roots, radicle and stem, but low expression value in leaves, the relatively low expression value was also detected in leave tissues when cold processing (Figs. 5, 6, Tables S8 and S9). On the other hand, in Group IVb, *Arabidopsis* laccase AtLAC15 and eggplant laccase SmLAC21 were clustered together (Fig. 2), AtLAC15 has been reported to be associated with oxidative polymerization of flavonoids in *Arabidopsis* seed coat (Pourcel et al., 2005), thus, SmLAC21 is supposed to have similar functions in eggplant. Finally, 47.9% of SmLACs could not be classified temporarily due to low homology with *Arabidopsis* laccases, thus, these laccases were temporarily named as Group VII and Group VIII (Fig. 2). From the above results, it could be predicted that *SmLACs* may function in multiple physiological processes, such as development, morphogenesis, and response to abiotic stress. Therefore, *SmLAC* gene expression should be investigated in response to multiple abiotic stresses by qRT-PCR analysis. Our results have suggested that 6 selected genes except for *SmLAC12* can be significantly induced by one or multiple abiotic stresses (Fig. 7), which indicates that *SmLACs* may function in response to various stress treatments. Further analysis has showed that *SmLAC12* gene is significantly inhibited by cold, drought and salt stress, while *SmLAC15* gene is significantly activated by the three treatments mentioned above, but plenty of efforts still should be further made to reveal the detailed biological function of genes *SmLAC12* and *SmLAC15*. Consequently, the two genes would be the focus of subsequent research on improving resistance of eggplant.

Analysis of gene structure and protein motif has showed that the most closely related members in the phylogenetic tree include similar exon/intron organization and common motif compositions (Fig. 3), suggesting the existence of a possible functional similarities among these closely related SmLAC proteins. However, within the eight basic groups, exon/intron organization and motif compositions were highly distinct (Fig. 3), which indicate that functional differentiation may present in each group of the eggplant laccase gene family. Further gene duplication analysis of *SmLAC* genes has indicated that the gene duplications happened from 8.27 Mya to 391.86 Mya (Table S6). Moreover, these genes have evolved in the selection of strong purification, based on the Ks value. In addition, the physical locations of *SmLAC* genes have suggested that tandem repeats are the main mechanisms of laccase gene duplication (Fig. 1). This result is basically consistent with the research result previously reported in soybean laccase family members (Wang et al., 2019). Furthermore, 21 *SmLAC* genes were collinear with *LAC* genes from tomato, *Arabidopsis* or rice, but none of *SmLAC* genes were collinear with *LAC* genes in other three species (Fig. 4, Table S7). This result reveals that gene duplication events may be greatly beneficial to the amplification of laccase gene family in eggplant and other species (Wang et al., 2019; Zhang et al., 2019).

## Conclusions

In this research, we identified 48 eggplant laccase members. Among them, 42 *SmLAC* gene members were unevenly distributed on 12 chromosomes and six *SmLAC* genes were associated with scaffolds, and only 25 SmLACs were divided into six groups, through multiple sequence alignments of 17 *Arabidopsis* laccases and 48 eggplant laccases. The number of exons contained in *SmLAC* genes ranged from one to 13, and the identified SmLACs included the conserved motif 1, motif 2, motif 3, motif 4, motif 5 and motif 10. Among these laccases from eggplant, eight pairs of tandem replications were identified. Moreover, all of these gene pairs were evolved in the selection of strong purifying. Further analysis showed that twenty-four pairs of collinearity between twenty-one *SmLAC* genes and twenty-four *SlLAC* genes, six pairs of collinearity between six *SmLAC* genes and six *AtLAC* genes, but only two pairs of collinearity between one *SmLAC* gene and two *OsLAC* genes. Twenty-one *SmLAC* genes were collinear with *LAC* genes from *Arabidopsis*, tomato or rice, but none of *SmLAC* genes were collinear with *LAC* genes in other three species. Furthermore, all *SmLAC* promoter regions were predicted to contain light responsive elements, indicating important roles in eggplant morphogenesis. Most of *SmLAC*s were forecasted to include cis-elements associated with ABA and AAI. Many *SmLACs* were predicted to involve in phytohormone responses (AUX, GA, Me-JA and SA) and stress responses (drought, defense and stress), a few may involve in low temperature stress or the development of meristem, endosperm and zein metabolism, and several maybe in relation with flavonoid biosynthetic, wound stress and the development of seed. In addition, based on RNA-seq data reported in previous study, expression patterns of *SmLACs* were analyzed in vegetative and reproductive organs, at different developmental stages, gene *SmLAC19* showed no expression in all of the examined developmental stages, seventeen *SmLAC* genes were ubiquitously expressed in all eggplant organs/tissues, but differentially expressed in different tissues, other thirty-one *SmLAC* genes showed relatively high levels in some organs, but relatively low levels or no-expression in many tissues/organs. Based on our unpublished RNA-seq data under cold stress, compared with normal growth conditions, cold stress could not significantly affect the expression of laccase family genes. But further qRT-PCR analysis showed that *SmLAC* genes selected can be significantly up-regulated or down-regulated in response to one or multiple stresses (cold, drought and salt stress).

## Supplemental Information

10.7717/peerj.12922/supp-1 Supplemental Information 1 Primers used in Real-Time PCR.

10.7717/peerj.12922/supp-2 Supplemental Information 2 Coding sequences of laccase family members in eggplant.

10.7717/peerj.12922/supp-3 Supplemental Information 3 Annotation information and peptide sequences of laccase family members in eggplant.

10.7717/peerj.12922/supp-4 Supplemental Information 4 Information of 48 SmLAC genes (protein length, molecular weight, isoelectric point, CDS length and Cell location).

10.7717/peerj.12922/supp-5 Supplemental Information 5 The 2.0 kb upstream sequence of the start codon (ATG) of each *SmLAC* gene.

10.7717/peerj.12922/supp-6 Supplemental Information 6 Tandem duplications of SmLAC gene pairs in eggplant and inference of duplication time.

10.7717/peerj.12922/supp-7 Supplemental Information 7 Synteny analysis between eggplant and other species.

10.7717/peerj.12922/supp-8 Supplemental Information 8 Expression values (FPKM) of 48 SmLAC genes from 18 eggplant samples. For fruit stage B and C, skin and pul tissues were put together.

10.7717/peerj.12922/supp-9 Supplemental Information 9 Expression values (FPKM) of 48 SmLAC genes under cold stress for 12h.

## Additional Information and Declarations

### Data Availability

The following information was supplied regarding data availability: The data is available at NCBI SRA: SRR15036047, SRR15036048, SRR15036049, SRR15036050.
